# Severe homocysteinemia in two givosiran-treated porphyria patients: is free heme deficiency the culprit?

**DOI:** 10.1007/s00277-021-04547-3

**Published:** 2021-05-29

**Authors:** Petro E. Petrides, Michael Klein, Elfriede Schuhmann, Heike Torkler, Brigitte Molitor, Christian Loehr, Zahra Obermeier, Maria K. Beykirch

**Affiliations:** 1 EPNET Center Munich, Hematology Oncology Center, University of Munich Medical School, Zweibrückenstr.2, 80331 Munich, Germany; 2 Klinikum Vest, Dorstener Strasse 151, 45657 Recklinghausen, Germany; 3 Homocysteine Laboratory, Labor Becker und Kollegen, Führichstr.70, 81671 Munich, Germany; 4 Genetics Laboratory, MVZ Eberhard, Brauhausstr.4, 44137 Dortmund, Germany; 5 Eurofin Laboratories, Rotthauser Str 19, 45879 Gelsenkirchen, Germany; 6 Department of Radiology, Klinikum Vest, Dorstener Strasse 151, 45657 Recklinghausen, Germany

**Keywords:** Acute porphyria, Givosiran, ∂-ALA-Synthase 1, Severe homocysteinemia, Free heme, N-homocysteinylation

## Abstract

Givosiran is a novel approach to treat patients with acute intermittent porphyrias (AIP) by silencing of ∂-ALA-synthase 1, the first enzyme of heme biosynthesis in the liver. We included two patients in the Envision study who responded clinically well to this treatment. However, in both patients, therapy had to be discontinued because of severe adverse effects: One patient (A) developed local injection reactions which continued to spread all over her body with increasing number of injections and eventually caused a severe systemic allergic reaction. Patient B was hospitalized because of a fulminant pancreatitis. Searching for possible causes, we also measured the patients plasma homocysteine (Hcy) levels in fluoride-containing collection tubes: by LC–MS/MS unexpectedly, plasma Hcy levels were 100 and 200 in patient A and between 100 and 400 μmol/l in patient B. Searching for germline mutations in 10 genes that are relevant for homocysteine metabolism only revealed hetero- and homozygous polymorphisms in the MTHFR gene. Alternatively, an acquired inhibition of cystathionine-beta-synthase which is important for homocysteine metabolism could explain the plasma homocysteine increase. This enzyme is heme-dependent: when we gave heme arginate to our patients, Hcy levels rapidly dropped. Hence, we conclude that inhibition of ∂-ALA-synthase 1 by givosiran causes a drop of free heme in the hepatocyte and therefore the excessive increase of plasma homocysteine. Hyperhomocysteinemia may contribute to the adverse effects seen in givosiran-treated patients which may be due to protein-N-homocysteinylation.

## Introduction

Acute porphyrias are inborn errors of heme metabolism which are characterized by abdominal attacks and neurological disturbances in symptomatic patients [1]. Therapy of choice for the acute attack is heme arginate [2]. A small percentage of porphyria patients suffer from repeated attacks which over time are more and more difficult to treat [3].

Since there is a medical need, new therapies are being developed which range from enzyme substitution therapy over mRNA application to silencer RNA (siRNA) approaches [4–6]. The latter has been most successful with the recent completion of the phase 3 study and subsequent approval of givosiran which suppresses ∂-aminolevulinic (ALA)-synthase 1, the first enzyme of heme biosynthesis [7].

In the liver, heme is not only important for electron transfer systems in mitochondria (such as cytochrome b5) but also—just to name a few—for various enzymes such as the family of cytochrome P450 enzymes, catalase, tryptophan pyrrolase, or cystathionine-ß-synthase, an important enzyme for homo-cysteine degradation.

Moreover, in recent years, new roles of the so-called free heme as a signal transducer and sensing site for gases such as O_2_, NO, or CO have been identified [8].

Inhibition of ∂-ALA-synthase 1 by the siRNA approach may partially impair heme biosynthesis. One investigation has focused on a potential influence of givosiran treatment on CYP450 enzymes (but not on other heme-dependent enzyme systems) and found only a moderate if any undesirable influence on CYP1A2, 2D6, 3A4, and 2C19 [9].

Here, we report that in givosiran-treated patients, the drug can severely impair homocysteine degradation leading to excessively high plasma homocysteine levels.

## Methods

### Study and patients

#### Study

In the study, acute porphyria patients could be included which had suffered from recurrent attacks (at least two within 6 months prior to inclusion). The study drug givosiran was given monthly at a subcutaneous dosage of 1.25 or 2.5 mg/kg body weight depending upon study amendments. During the first 6 months, patients were either on placebo or study drug; after that, all patients received givosiran.

#### Patients

Patient A is a female patient who was diagnosed with AIP; she had been diagnosed at the age of 30 years. Her PBG-deaminase activity was reduced to 61%; mutation analysis had revealed a heterozygous pArg 149* mutation in hydroxymethylbilane synthase (HMBS) (a nonsense mutation in one allele of her PBGD-gene). She had suffered from recurrent attacks which required the application of heme arginate.

At the age of 37 years, she was included in the study. Prior to inclusion, her urine ∂-ALA and PBG values were 546 and 239 μmol/g creatinine, resp.

Patient B is a female patient in whom AIP had been diagnosed at the age of 28 years. Her porphobilinogen (PBG)-deaminase activity was reduced to 48%; the genetic analysis had revealed a nonsense mutation pTyr46* in the HMBS gene. Over several years, she had been suffering from recurrent attacks which had been treated with contraceptives, leuprorelin, and prophylactic heme arginate. At the age of 31 years, she was included in the study. Prior to the study enrolment, her urine ∂-ALA and PBG urine values were 352 and 293 μmol/g creatinine, resp.

Both patients are part of our Munich cohort [3] but were also treated in local hospitals.

#### Plasma homocysteine determination

Plasma homocysteine was determined by liquid chromatography/tandem mass spectroscopy (LC/MS/MS) [10]. Blood samples were collected in Becton Deckinson special tubes (BD vacutainer 367764) containing sodium heparin (28 U/ml) and fluoride (4 mg/ml). In these tubes, homocysteine is stable at room temperature for 3 days [11]. Prior to analysis, the plasma samples were reduced with dithiothreitol (DTT) to produce homocysteine from disulfides. Protein-bound homocysteine was then released through denaturation and precipitation. Homocysteine is further resolved by reverse phased chromatography and analyzed by LC/MS/MS. A stable isotope-labeled internal standard is used for quantification.

In some instances, homocysteine determination was carried out with a competitive immunoassay luminescence test (ADVIA CENTAUR® Siemens) which is more rapid but less reliable at homocysteine values above 100 μmol/l [12]. Homocysteine is enzymatically converted into S-adenosyl-homocysteine (SAH) which is quantified in a competitive immunoassay (labelled anti-SAH antibody: magnetic particles coupled with SAH). Samples with higher homocysteine values were measured again with the LC/MS method.

#### Next-generation sequencing of genes involved in homocysteine metabolism

Patient samples were analyzed after written informed consent by next-generation sequencing (NGS) using a panel of targeting of the following ten genes:

ABCD4 (ATP-binding cassette, subfamily D, member 4), CBS (cystathionine-beta-synthase), LMBRD1 (LMBR1 domain-containing protein 1), MMACHC (metabolism of cobalamin-associated C), MMADHC (MMADHC gene), MMUT (methylmalonyl-CoA mutase), MTHFR (methylenetetrahydrofolate reductase), MTR (5-methyltetrahydrofolate homocysteine S-methyltransferase), MTRR (methionine synthase reductase), and PRDX1 (peroxiredoxin 1).

NGS was performed using the Twist Hybrid Capture system in combination with MiSeq instrument (Illumina, San Diego, CA).

## Results

### Clinical course of the two patients

**Fig. 1:**
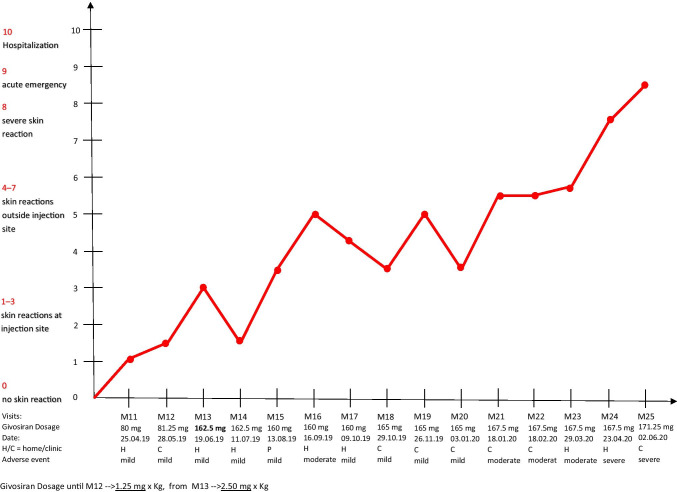
Development of skin reactions over time in patient A upon treatment with givosiran

Patient A was enrolled in the Envision study in June 2018 and reached the verum phase in November 2018. For the first couple of months she was given 1.25 mg givosiran monthly, at injection 13, the dose was adjusted to 2.5 mg. After good tolerance initially, she developed local skin reactions at the injection site, steadily increasing from injection to injection. She also reported a swelling of her hands and feet (Fig. 1).

She had no abdominal symptoms so she did not require any heme arginate infusions. At month 16, skin reactions started to spread all over her body with the increasing number of injections and eventually resulted in a severe systemic allergic reaction.

**Fig. 2:**
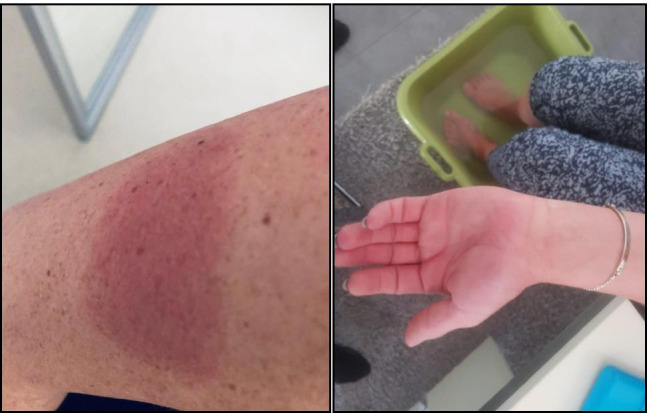
Skin reactions in patient A at injection no 25

Because of this development, injection 25 was not given at home but in the clinic and split into two parts: despite the antiallergic premedication with cetirizine (10 mg per day), the patient showed immediately after the injection an even more pronounced reaction with shaking chills, chest tightness, and acute dyspnea. Her heart rate went up to 166/min. Her blood pressure was not measurable because of the chills. She also developed acute erythrodermia in the face, neckline, and both arms as well as swelling of her hands (Fig. 2). Urticarial reactions subsequently appeared at both injection sites. She was given an injection of dimethindene maleate and recovered over the next 3 h. Upon laboratory testing, she had a normal blood cell count (leukocytes, hemoglobin, platelets) but MCV was slightly elevated (105 μm^3^ (normal range 80–100)). Immunoglobulins were normal except slight elevations of IgA (4.15 g/l (normal 0.7–4.0)) and IgE (180 (normal < 100 U/ml)). Several liver enzymes were also slightly elevated (aspartate amino transferase 79 U/l (normal < 35), glutamine pyruvate transaminase 51 U/l (normal < 35), y-glutamyl transferase 217 U/l (normal < 40), and alkaline phosphatase (AP) 148 U/l (normal 35–105)). Glutamate dehydrogenase (GLDH), however, was strongly elevated to 79.1 U/l (normal < 5) which indicates a toxic liver damage since the enzyme is only located in the mitochondria. Ferritin had always been above normal (422 μmol/l (normal < 100)).

Within the normal range were creatinine (0.8 mg/ml); vitamins (B12: 237 μg/l (normal range 187–883); folic acid: 4.4 μg/l (normal range 2.3–17.6); and B6: 6.2 μg/l (normal range 5–30)); and tryptase, C1-esterase-inhibitor activity, C3/C4 complement, and C-reactive protein.

Patient B was enrolled in July 2018. She needed heme arginate during the first 6 months, indicating that she had been randomized to the placebo arm. After switching to the givosiran arm in January 2019, she showed a dramatic clinical improvement.

**Fig. 3:**
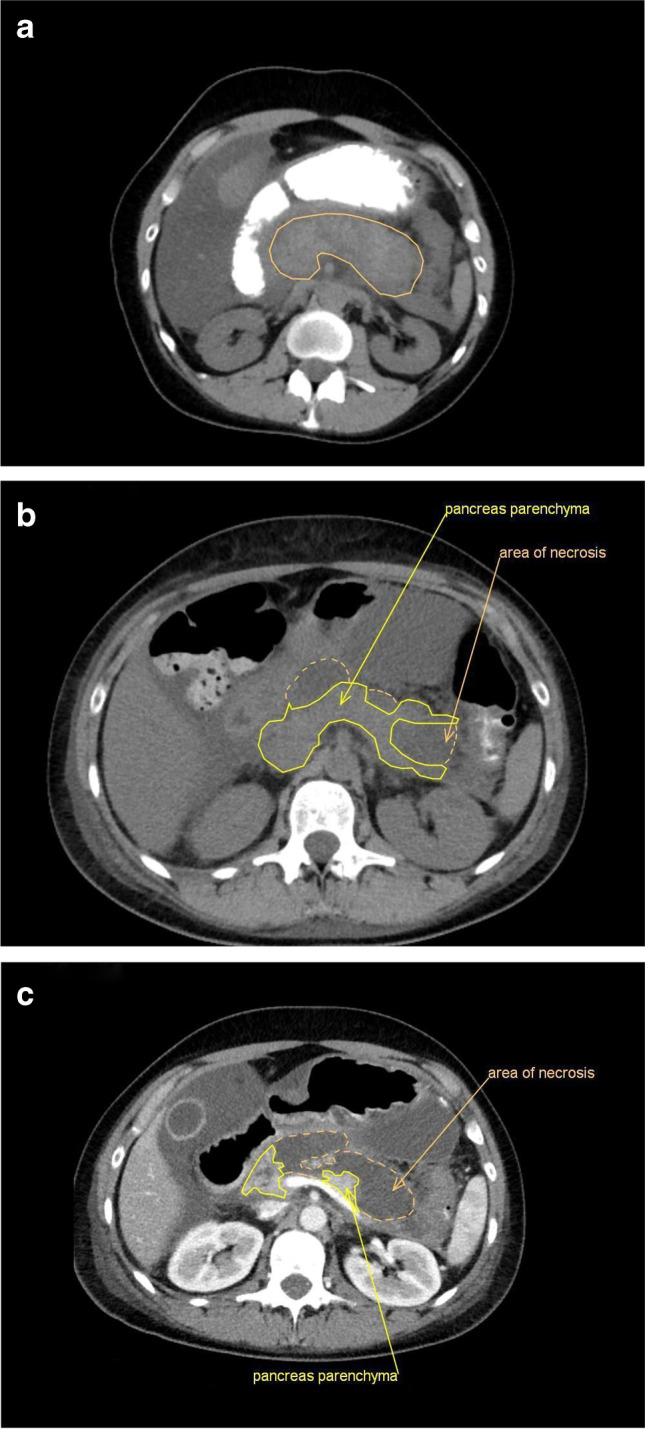
**a** Native abdominal CT scan with oral contrast dye at the day of admission: acute edematous pancreatitis (border of the pancreas represented by solid line) with significant reaction of the surrounding tissue representing beginning necrotic streets. **b** Native abdominal CT scan 8 days later: no clear signs of pancreatic necrosis (dashed line) with ongoing strong necrotic streets. **c** Abdominal CT scan after intravenous contrast dye another 2 days later: farther pronounced and progressive necrotic streets, increasing pancreas necrosis with less and less normal parenchyma left

Her treatment was uneventful until injection no. 24 when she experienced severe abdominal pain. Upon hospital admission (27 April 2020) 5 days later, a computerized tomography (CT) scan showed an acute necrotizing pancreatitis and pseudocysts (Fig. 3). Parenteral nutrition (including vitamins and antibiotics) was started, and the implantation of a gastrointestinal (GI) stent was performed in order to empty the cyst. Upon admission, she had had leukocytosis (37,300/μl, normal < 10,000), a high C-reactive protein (59.7 mg/l, normal < 5), and a high lipase (2378 U/ml, normal < 60) and ferritin values (> 2000 ng/ml normal < 100). Creatinine value elevated to 2.1 mg/dl (normal < 1.0).

Vitamin B12 was with 473.2 pg/ml in the normal range (197–771), but folic acid slightly diminished (2.4 (normal 4.8–37.3) ng/ml).

Upon treatment, leukocytes rapidly fell to 8000/μl, lipase to 43 U/ml, and creatinine to 0.62 mg/dl, but CRP remained elevated at 51.1 mg/l.

After the stent removal from the stomach, she retained a stable clinical condition so that she could be dismissed from the hospital (15 May 2020).

Two weeks later (end of May 2020), she presented herself again in the emergency room and had to be readmitted for an infected pseudocyst: she was treated with antibiotics and remained hospitalized for 2 months because of additional problems such as temporary diabetes mellitus and partial adrenal insufficiency.

She had been on low-dose olanzapine (5 mg daily) for the treatment of opiate-induced nausea for 3 years. After the first hospital admission, olanzapine was discontinued. Later, she was rechallenged to the drug in the clinic without any problems which was discontinued because of lack of efficacy.

## Plasma homocysteine measurements in both patients

Since two groups [13, 14] had reported that plasma homocysteine can be elevated in patients with acute porphyrias, we also measured plasma homocysteine levels by LC/MS/MS in our patients: unexpectedly, the first homocysteine level measured was 113 μmol/l in patient A (at the day of the severe drug reaction) and 408 μmol/l in patient B (after recovery from the pancreatitis). Homocysteine plasma levels were followed up and remained consistently high (increasing to 136 μmol/l in patient A), until they declined 3 months after discontinuation of givosiran.

In patient A, plasma methionine was also determined and shown to be elevated (9.5 mg/l, normal range 0.7–6.0) indicating an impairment of homocysteine degradation.

In both patients, creatinine and vitamin levels (B12, B6, and folic acid) were normal at the time of first homocysteine measurement.

## NGS sequencing of homocysteine metabolism genes

In order to rule out any mutations in genes involved in homocysteine metabolism, ten genes in the DNA of both patients were analyzed: except for the MTHFR gene, no mutation was found in either patient. Analysis of patient A revealed a heterozygous mutation (polymorphism) and patient B a homozygous mutation (polymorphism) in position 677 (C > T) of the MTHFR gene.

## Homocysteine plasma levels after heme arginate infusion

Heterozygosity of the MTHFR variant C677T usually causes only mild if any elevations of plasma homocysteine; homozygosity of the MTHFR variant C677T is associated with elevations up to 100 μmol/l, indicating additional reasons for the increase. Since the search for inborn mutations in other genes relevant for the metabolism of homocysteine did not yield to any results, we considered other explanations such as acquired metabolic changes. Homocysteine is also degraded by the enzyme cystathionine-ß-synthase (CBS), which is a pyridoxal-phosphate (PALP)-dependent enzyme, but also heme-dependent [15]. If a diminished production of heme would cause a loss of CBS activity, this could contribute to an increase of the level of homocysteine. To test this hypothesis, both patients received treatment with heme arginate (Normosang®) for several days, which lowered not only ∂-ALA and PBG (data not shown) but also decreased homocysteine plasma levels. Sometime later, however, the levels were up again and even higher than before the heme arginate infusion (Table 1).

**Table 1:** Response of plasma homocysteine levels (μmol/l) in patients A and B to the infusion of Normosang® (1 ampoule (containing 250 mg heme arginate = X) per day dissolved in saline containing human serum albumin)

Patient A	15/06/20*	18/06/20	19/06/20	22/06/20	02/07/20
Heme arginate	X	X	X	X	
Homocysteine	130	34	49		320
Patient B	30/06/20**	01/7/20	02/07/20		05/08/20
Heme arginate	X	X	X		
Homocysteine	87	33	23		127

*Last givosiran injection 02.06.2020, **last givosiran injection 22.4.2020.

### Follow-up of the patients after study discontinuation until March 2021

Patient A remained stable without requiring further heme arginate therapy. Her plasma homocysteine levels continuously decreased over several weeks (127 > 85 > 66 > 22 μmol/l).

Patient B suffered from recurrent attacks which made the repeated weekly application of heme arginate necessary.

Moreover, at the end of October 2020, she was hospitalized again for 4 weeks because of a colitis.

In February 2021 (until present), she was readmitted to the hospital because of a mechanical ileus and underwent abdominal surgery to treat a stenosis in the sigma region. Histological analysis revealed neither chronic inflammation nor infection but massive fibrosis of the resected intestine.

Upon follow-up, vitamin levels were normal (folic acid 10.37 ng/ml, vitamin B12 681.8 pg/ml, and vitamin B6 17.2 μg/l).

Her plasma homocysteine levels also gradually declined over several weeks (59.9 > 37.4 > 17.5 > 24.5 μmol/l).

## Discussion

Heme arginate is still the therapy of choice for patients with acute porphyria attacks [2]. It can also be used prophylactically [16]. The drug is, however, only partially efficacious in the small group of patients with recurrent attacks [3]. Hence, there is a medical need to develop novel drugs based upon enzyme replacement through recombinant enzyme technology, induction of enzyme production by mRNA technology, recovery of enzyme functionality [17], or inhibition of ∂-ALA-synthase-1 by targeting specifically the mRNA by a synthetic chemically modified double-stranded small interfering ribonucleic acid (siRNA).

Utilizing the latter principle, givosiran is a novel innovative therapy which has been shown to be effective and safe in a phase 3 trial [7] which led to the registration in the USA and EU. Major adverse effects reported in this trial were renal function impairment in 15%, injection site reactions (25%), and rash (6%) as well as increase of alanine aspartate transferase (8%).

We have treated two female patients with recurrent attacks within the Envision study. After initial excellent clinical response, both patients developed adverse effects (whole body skin rash or pancreatitis, resp.) which unfortunately led to the discontinuation of the therapy with givosiran.

When looking for possible explanations, we unexpectedly observed extreme elevations of the plasma homocysteine levels in both these patients.

Plasma homocysteine elevations in patients with acute porphyrias have first been reported by To-Figueras a decade ago [13]: they found homocysteine elevations up to 80 μmol/l in 24 patients with AIP when compared to healthy controls. In one of these patients, they measured plasma homocysteine before and after repeated heme arginate infusions and observed a reduction by the infusion but a rapid recovery at the initiation of the next session. Although they observed low levels of vitamin B6 in their cohort, they could not correlate normal and lower B6 levels in the patients with hyperhomocysteinemia. Alternatively, they are discussing a reduction of cystathionine-ß-synthase activity which is not only a B6 (pyridoxal 5′-phosphate PLP) but also heme-dependent enzyme [15].

The studies were recently extended by Ventura and coworkers [14]. They investigated 46 acute porphyria (AP) patients which they divided in three groups: symptomatic patients (AP-SP), patients with biochemical alterations (AP-BA), and asymptomatic carriers (AP-AC). On average, symptomatic patients have higher plasma homocysteine values (27.6 μmol/l with the highest value measured being 80 μmol/l) than AP-BA and AP-AC individuals (17.1 or 10.7 μmol/l, resp.). Since they found lower B6 levels in their cohort as compared to healthy Italian subjects, they hypothesized that the induction of ∂-ALA-synthase-1 may redirect B6 from other metabolic pathways such as B6-dependent homocysteine metabolism and by this cause hyperhomocysteinemia.

When we decided to analyze plasma homocysteine in our patients, we expected normal or slightly elevated levels since both patients had clinically responded well to the treatment with givosiran. However, we observed levels which in both patients exceeded 100 μmol/l.

Severe homocysteinemias (between 100 and 500 μmol/l) are usually not seen in adults but only observed in newborns with inborn cystathionine-ß-synthase (CBS) defects [18]. If left untreated, these patients develop developmental impairment as well as thromboembolic complications (such as stroke), pancreatitis, or skin alterations.

Looking for inborn errors of homocysteine metabolism in our patients, we identified a heterozygous mutation at position c.677 (C > T) of the MTHF-reductase gene in patient A and a homozygous variant in patient B. However, individuals with these polymorphisms usually show plasma homocysteine levels only up to 50 and on very rare occasions up to 100 μmol/l.

NGS sequencing of nine other genes relevant for the homocysteine metabolism (ABCD4, CBS, LMBRD1, MMACHC, MMADHC, MMUT, MTR, MTRR, and PRDX1) did not reveal any additional mutated genes.

Homocysteine is degraded by CBS which in contrast to other enzymes involved in amino acid metabolism not only requires vitamin B6 but, in addition, also heme for its full activity (Fig. 3). CBS not only regulates homocysteine metabolism but also contributes to the biosynthesis of the gaseous transmitter H_2_S through which it is involved in cellular energetics, redox status, DNA methylation, and protein modification [19]. Moreover, through the transsulfuration pathway, the production of cysteine and the antioxidant glutathione are regulated [20]. In CBS, heme is not directly involved in the catalytic mechanism but guarantees the three-dimensional folding of enzyme mandatory for enzyme activity [21] and also serves as a signal molecule.

**Fig. 4:**
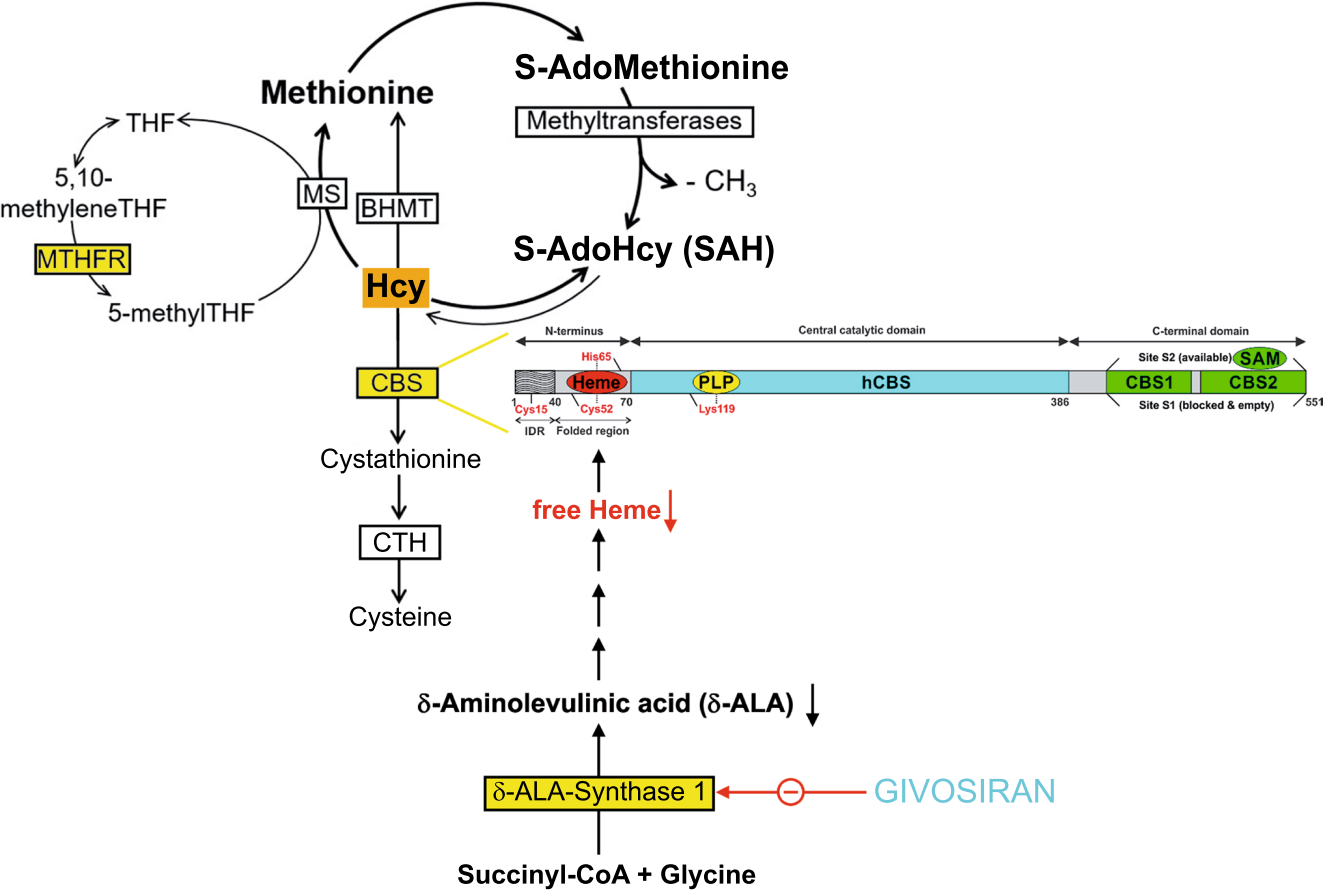
Simplified diagram of homocysteine metabolism, domain structure of cystathionin-ß-synthase, and mechanism of action of givosiran. Homocysteine can be removed either through cystathionin-ß-synthase (CBS) or by its remethylation to methionine: this remethylation can be folate-dependent (requiring the activities of methionine synthase (MS) and 5,10-methylentertahydrofolate reductase (MTHFR)) or folate-independent (betaine-homocysteine methyltransferase (BHMT)). CBS is a heme-dependent enzyme: Residues 40–70 form a folded region, which binds heme cofactor, axially ligated by residues CYS52 and HIS65. Givosiran inhibits the production of ∂-ALA-synthase 1 which reduces the generation of ∂-ALA and heme (modified after [39] and [45])

Thus, we hypothesized that givosiran causes an acquired CBS deficiency through depletion of the heme pool which leads to a disturbance of homocysteine degradation aggravated by concomitant hetero- or homozygous MTHFR polymorphisms (Fig. 4).

When we gave heme arginate to both our patients, plasma homocysteine levels rapidly fell but increased again similar to what had been observed previously [13]. Only after disappearance of the activity of givosiran which lasts at least 3 months [22], they fell to fluctuating values in the moderately elevated range.

We have reported these results to the sponsor of the study as well as all other Envision study centers [23]. Meanwhile, strongly elevated plasma homocysteine levels have also been observed in other porphyria centers [24]. Moreover, in a study on frozen samples from the Envision study patients, Alnylam has reported elevated plasma homocysteine levels, however, without providing any further details [25].

Impairment of CBS activity may not be the only consequence of heme deficiency but could even be the tip of an iceberg: other heme-dependent metabolic pathways such as tryptophan or testosterone degradation may also be involved [26]. Moreover, in recent years, in addition to the prototypical functions of oxygen metabolism, electron transfer, and CYP450 activities, a plethora of new roles of a mobile (also called free or regulatory) heme in signal transduction has been elucidated [27]. Hence, only metabolomic studies will help to find out which metabolic pathways are being influenced by givosiran treatment [28].

In addition, side effects of givosiran such as renal impairment may not be a direct effect but may also be caused by disturbed hepatic metabolism with secondary effects on the kidney [29].

Recently, Nakajima′group in Japan has developed an experimental knockdown model making mice heterozygous for ∂-ALA-synthase 1 [30]. This model resembles the siRNA approach: in their mice, the authors have observed a reduction of free, but not total heme in hepatocytes and also impaired glucose intolerance and insulin resistance [31].

To which extent severe homocysteinemia (levels above 100 μmol/l) has caused or contributed to the adverse effects observed in our two patients cannot be decided at present since such high values occur only in newborns with inborn homocystinuria. Hence, most studies in adults are being carried out on patients with intermediate (31–100) or moderate (16–30) plasma homocysteine levels. However, pancreatitis is a common feature in patients with inborn homocystinuria [18, 32, 33]. Moreover, hyperhomocysteinemia has been associated in several studies with acute and chronic pancreatitis [34–37]. Alternatively, olanzapine taken by our patient (B) can also cause pancreatitis [38]. Our patient, however, was on a low dose without any problems for 3 years and later reexposed to the drug without exacerbation of the pancreatitis.

Givosiran can cause local injection reactions and rash as reported in the Envision study [7]. Excessive homocysteinemia may aggravate these side effects through the induction of endothelial dysfunction in the skin [39, 40] and mitochondrial dysfunction [41] in the liver (see above). Part of these effects might be due to a N-homocysteinylation of various proteins [42, 43].

Interestingly enough, fibrosis of the intestinal tract—as seen in patient B—is promoted by homocysteine in an experimental animal model [44].

From our observations, we conclude that givosiran aggravates the disturbance of homocysteine metabolism which is already present in many patients with acute porphyrias. Mechanism of action may be a reduction of free heme in the hepatocyte. The degree of further homocysteine elevation will be determined (provided that renal function and vitamin status are normal) by concomitantly present polymorphisms of the MTHFR gene. Since givosiran therapy is a continuous therapy, chronic severe homocysteinemia may lead to various long-term complications.

In order to avoid these, we recommend that all patients who are considered for therapy with givosiran should be assayed for plasma homocysteine levels prior to initiation of therapy and while being on therapy. In case of pretherapeutical homocysteinemia, patients should be tested for vitamin B6, folic acid, and vitamin B12 levels as well as for MTHFR polymorphisms. Depending upon the results, vitamin replacement therapy (possibly also with betaine) should be implemented.

Further studies will be needed to reveal whether the current monthly treatment with givosiran has to be modified.

## Abbreviations

∂-ALA: ∂-Aminolevulinic acid
PBG: Porphobilinogen
Hcy: Homocysteine
AIP: Acute intermittent porphyria
GLDH: Glutamate dehydrogenase
MTHFR: Methylenetetrahydrofolate reductase
CYP450: Cytochrome P450
CBS: Cystathionine-beta-synthase
LC/MS/MS: Liquid chromatography/tandem mass spectroscopy
siRNA: Silencer RNA
DTT: Dithiothreitol
SAH: S-adenosylmethionine
NGS: Next-generation sequencing
